# Buzz-Pollinated Crops: A Global Review and Meta-analysis of the Effects of Supplemental Bee Pollination in Tomato

**DOI:** 10.1093/jee/toab009

**Published:** 2021-02-22

**Authors:** Hazel Cooley, Mario Vallejo-Marín

**Affiliations:** 1 Department of Biological and Environmental Sciences, University of Stirling. Stirling, Scotland, UK; 2 School of Natural and Environmental Sciences, Newcastle University, Newcastle upon Tyne, UK

**Keywords:** agriculture, bee, buzz pollination, tomato, pollinator

## Abstract

Buzz-pollinated plants require visitation from vibration producing bee species to elicit full pollen release. Several important food crops are buzz-pollinated including tomato, eggplant, kiwi, and blueberry. Although more than half of all bee species can buzz pollinate, the most commonly deployed supplemental pollinator, *Apis mellifera* L. (Hymenoptera: Apidae; honey bees), cannot produce vibrations to remove pollen. Here, we provide a list of buzz-pollinated food crops and discuss the extent to which they rely on pollination by vibration-producing bees. We then use the most commonly cultivated of these crops, the tomato, *Solanum lycopersicum* L. (Solanales: Solanaceae), as a case study to investigate the effect of different pollination treatments on aspects of fruit quality. Following a systematic review of the literature, we statistically analyzed 71 experiments from 24 studies across different geopolitical regions and conducted a meta-analysis on a subset of 21 of these experiments. Our results show that both supplemental pollination by buzz-pollinating bees and open pollination by assemblages of bees, which include buzz pollinators, significantly increase tomato fruit weight compared to a no-pollination control. In contrast, auxin treatment, artificial mechanical vibrations, or supplemental pollination by non-buzz-pollinating bees (including *Apis* spp.), do not significantly increase fruit weight. Finally, we compare strategies for providing bee pollination in tomato cultivation around the globe and highlight how using buzz-pollinating bees might improve tomato yield, particularly in some geographic regions. We conclude that employing native, wild buzz pollinators can deliver important economic benefits with reduced environmental risks and increased advantages for both developed and emerging economies.

Understanding which pollinator groups are best suited to pollinate food crops is imperative for optimizing the yield and quality of agricultural crops worldwide. The production of roughly 35% of the food we eat is dependent on animal pollination services (Potts et al. 2016). Insect pollinators are frequently deployed in agricultural settings in an attempt to supplement natural pollinators and to increase the yield and quality of agricultural produce (Velthuis and Van Doorn 2006, Rucker et al. 2012). Globally, supplemental crop pollination services are predominantly provided by a handful of bee species, namely honey bees (*Apis mellifera*) and, to a lesser extent, by some bumblebee species (e.g., *Bombus terrestris* L., *Bombus impatiens* Cresson and *Bombus ignitus* Smith; Hymenoptera: Apidae), and stingless bees (Hymenoptera: Apidae: Meliponini). However, supplemental bee pollinators differ in their ability to pollinate different crops, and the deployment of a bee species ill-suited to a given crop, reduces their pollination services (Greenleaf and Kremen 2006, Macias-Macias et al. 2009, Benjamin and Winfree 2014). Therefore, identifying and capitalizing upon the characteristics that make some pollinators better suited than others may considerably enhance crop yield and quality.

Buzz-pollinated crops may be particularly suitable to study the extent to which different pollinators affect crop yields. In buzz-pollinated plants, bee pollinators use vibrations generated by their thoracic muscles to efficiently remove pollen from flowers with specialized morphologies (Buchmann 1983, Vallejo-Marin 2019). Most buzz-pollinated plants lack nectar and rely on pollen provisions to attract and reward pollinators (Vallejo-Marin et al. 2010). Generally, in buzz-pollinated plants, pollen-storing anthers open through small apical pores or slits (poricidal anthers), from which pollen can be released in large quantities when vibrated by a pollinator (Buchmann 1983, Russell et al. 2017). Approximately half of all bee species use this type of vibration-assisted foraging (‘sonication’ or floral buzzing; Cardinal et al. 2018). However, the most important supplemental bee pollinator, the honey bee, is incapable of vibrating flowers to remove pollen (King and Buchmann 2003), and consequently may have a reduced effectiveness as a pollinator of buzz-pollinated plants.

Several important crops including tomato, eggplant (*Solanum melongena* L., Solanales: Solanaceae), kiwi (*Actinidia deliciosa* Chevalier, Liang & Ferguson, Ericales: Actinidiaceae), and blueberry (*Vaccinium* spp. L., Ericales: Ericaceae) are buzz pollinated (De Luca et al. 2013, Corbet and Huang 2014, Cardinal et al. 2018). For these crops, pollen-collecting visitors such as honey bees may be providing suboptimal services compared to other bees capable of buzz pollination, such as bumblebees and stingless bees. To date, no comprehensive review has attempted to summarize the effects of different types of floral visitors on the pollination of buzz-pollinated crops. The goal of this study is twofold: 1) To discuss the extent to which buzz-pollinated crops rely on buzz pollination for crop yield and quality and 2) to use tomato (*Solanum lycopersicum*), which is the best-studied buzz-pollinated crop, as a case study to conduct a meta-analysis of the effect of different types of pollination treatments on fruit yield. We place our findings on tomato pollination in the context of major geopolitical areas and discuss the implications of our findings for the pollination of agricultural crops, and the conservation of wild bees.

## Which Crops Are Buzz Pollinated?

Buzz pollination is a pollination syndrome in which bees use vibrations to pollinate flowers with specialized morphologies (Vallejo-Marin 2019, Pritchard and Vallejo-Marín 2020). It occurs in more than 20,000 species of flowering plants which have a diverse array of floral architectures (Buchmann 1983). Here, we define buzz-pollinated crops as those in which floral morphology limits access to pollen rewards and whose flowers require visitation by vibration-producing insects in order to achieve full seed set (Table 1). In most cases, the morphology restricting pollen access is the presence of poricidal anthers (e.g., tomato, eggplant, and kiwi; Buchmann 1983), but in some cases, as in blueberry flowers, narrow and bell-shaped corollas can aid in restricting pollen access to certain floral visitors (De Luca et al. 2013, Corbet and Huang 2014, Russell et al. 2017).

**Table 1.** Buzz-pollinated food crops consumed on global or regional scales

Common name(s)	Species	Family	Poricidal anthers	Pollinators commonly used	Place of origin	Scale of cultivation	Top producers	Annual economic export value	Global average price (per kg)	References
Kiwi	*Actinidia deliciosa*	Actinidiaceae	Yes	*Bombus, Apis,* wild pollinators	China	Large scale- global	China, Italy, New Zealand	USD $4.49B	USD $3.45	(Ferguson 1984, King and Ferguson 1994)
Tamarillo, Tree Tomato	*Solanum betaceum*	Solanaceae	Yes	*--*	Andes	Large scale - global	Colombia, South Africa	--	USD $1.66	(Ramírez and Kallarackal 2019)
Bush Tomato	*Solanum chippendalei*	Solanaceae	Yes	*--*	Australia	Small scale - regional	--	--	--	(Martine et al. 2013)
Wild Tomatoes	*Solanum habrochaites, S. chilense, S. oeucianum, S. peruvianum, S. pennelli, S. neorickii, S. chimelwskii*	Solanaceae	Yes	*--*	Ecuador, Peru	Small scale - regional	--	--	--	(Peralta and Spooner 2000)
Tomato	*Solanum lycopersicum*	Solanaceae	Yes	*Bombus, Melipona,* wild pollinators	Americas	Large scale- global	China, India, United States	USD $10.06B	USD $5.63	(De Luca and Vallejo-Marin 2013, Bergougnoux 2014)
Eggplant	*Solanum melongena*	Solanaceae	Yes	*Bombus, Melipona, wild pollinators*	India or Africa	Large scale- global	China, India, Egypt	USD $536.26M	USD $1.11	(Lester and Hasan 1991, Jayasinghe et al. 2017)
Pepino Dulce, Sweet Cucumber^*b*^	*Solanum muricatum*	Solanaceae	Yes	*--*	South America	Large scale – regional^*b*^	Chile	--	USD $1.07	(Anilkumar and Murugan 2014)
Currant Tomato	*Solanum pimpinellifolium*	Solanaceae	Yes	*--*	Ecuador	Small scale – regional	--	--	--	(Peralta and Spooner 2000)
Lulo de perro^*a*^	*Solanum pseudolulo*	Solanaceae	Yes	*--*	South America	Small scale – regional^*a*^	--	--	--	(Ramírez et al. 2018)
Lulo, Naranjilla^*a*^	*Solanum quitoense, Solanum septentrionale*	Solanaceae	Yes	*--*	South America	Large scale – regional^*a*^	Colombia, Ecuador	--	USD $2.26	(Ramírez et al. 2018)
Cocona	*Solanum sessiliflorum*	Solanaceae	Yes	*--*	South America	Small scale - regional	--	--	--	(Ramírez 2020)
Turkey Berry, Wild Eggplant, Pea Aubergine	*Solanum torvum*	Solanaceae	Yes	*--*	Americas	Small scale - regional	--	--	--	(Liu and Pemberton 2009)
Potato	*Solanum tuberosum*	Solanaceae	Yes	*None*	Peru	Large scale - global	China, India, Nigeria	$691.40M	USD $0.51	(Bradshaw and Ramsay 2009)
Toronjo, Conquina Melon^*a*^	*Solanum vestissimum*	Solanaceae	Yes	*--*	South America	Small scale – regional^*a*^	--	--	--	(Ramírez et al. 2018)
Lowbush Blueberry	*Vaccinium angustifolium*	Ericaceae	Yes	*Bombus, Apis, Osmia,* wild pollinators	Eastern North America	Large scale-global	--	--	--	(MacKenzie 2008, Retamales and Hancock 2018)
Rabbiteye Blueberry	*Vaccinium ashei*	Ericaceae	Yes	*Bombus, Apis, Osmia,* wild pollinators	South eastern United States	Large scale-global	--	--	--	(MacKenzie 2008, Retamales and Hancock 2018)
Highbush Blueberry	*Vaccinium corymbosum*	Ericaceae	Yes	*Bombus, Apis, Osmia,* wild pollinators	North America	Large scale- global	United States, Canada, Mexico	USD $3.73B	USD $9.05	(MacKenzie 2008, Retamales and Hancock 2018)
Cranberry	*Vaccinium oxycoccos, V. macrocarpon*	Ericaceae	Yes	*Bombus, Apis, Osmia,* wild pollinators	Northern regions of Europe, Asia and America	Large scale-global	United States, Canada, Chile	USD $3.73B	USD $4.37	(MacKenzie 2008, Retamales and Hancock 2018)

^*a*^Crops which have been identified as ‘of interest’ for investigation for future global agricultural food crops; ^*b*^crops where attempts are in progress to cultivate them outside of their native range. Top producers, annual economic export values, and global average price values were obtained from https://www.tridge.com. All other information can be found in references cited for each row.

Buzz-pollinated crops as defined above can be visited and pollinated to some extent by non-buzzing bees. Nevertheless, buzz-pollinating bees are often more efficient pollinators as shown in eggplant (Hikawa 2004), blueberry and cranberry (Stubbs and Drummond 1996, Javorek et al. 2002), kiwifruit (Pomeroy and Fisher 2002, Kim et al. 2005), and tomato (Banda and Paxton 1991). In blueberries, for example, honey bees can visit flowers to collect nectar (Javorek 2002), and these managed non-buzzing bees can be used to pollinate this crop (Martin et al. 2019). However, honey bees are inefficient pollinators of blueberries, requiring four times more visits to transfer the same amount of pollen compared to buzz-pollinating bees (Javorek 2002). The sheer abundance of honey bees in well stocked fields may, in some cases, balance their inefficiencies leading to adequate pollination (Lomond and Larson 1983, Aras et al. 1996, Dedej and Delaplane 2003, Martin et al. 2019). Yet, when honey bees are not abundant visitors to blueberries, visitation by wild bees, including buzz pollinators, improves fruit yield and fruit quality (Nicholson and Ricketts 2019). Honey bees in blueberry, tomato, and kiwi crops have also been observed to have a strong preference for competing flowering species and they are less faithful visitors to buzz-pollinated crops than buzz-pollinating bees (Sampson and Cane 2000, Stubbs and Drummond 2001, Pomeroy and Fisher 2002, Sabara et al 2004). The limited pollen rewards available to non-buzz pollinators compared to buzz pollinators (who receive more pollen per visit) might partially explain this reduced floral fidelity. Therefore, buzz-pollinated crops are likely to be more efficiently pollinated by bees that use vibrations to collect pollen from their flowers.

Although buzz-pollinated flowers require visitation by buzzing bees to reproduce, bees use vibrations to remove pollen from a variety of flowers, including non-buzz-pollinated plants (Russell et al. 2017). The production of floral vibrations is one of several behaviors available to some bees to efficiently collect floral resources. Several crops are occasionally buzzed by bees, but are not buzz-pollinated as defined above (Buchmann 1985, Russell et al. 2017). Importantly, pollen in these flowers can be easily accessed by bees without the need for mechanical vibrations. Examples include a variety of gourds and squashes (*Curcubita* spp. L. Cucurbitaceae: Cucurbitaceae), persimmon (*Diospyros virginiana* L., Ericales: Ebenaceae), and almond (*Prunus dulcis* Mill., Rosales: Rosaceae; Russell et al. 2017). While bee pollination may still be important in these crops, both buzzing bees and non-buzzing insects are capable of functioning as pollinators.

Plants with flowers typical of the buzz pollination syndrome are found in at least 64 plant families, many of which may contain plants from which humans derive useful products, materials, or foods. Identifying buzz pollination syndrome could therefore help optimize pollination, and potentially yield and quality, of a variety of important products (Buchmann 1983). Buzz-pollinated crops include major food plants such as tomatoes, kiwis, blueberries, cranberries, and eggplant (De Luca et al. 2013, Corbet and Huang 2014, Cardinal et al. 2018). Table 1 summarizes the food crops exhibiting the buzz pollination syndrome as defined above. Prominent among them, are plants in the genus *Solanum* where buzz pollination is well documented (De Luca and Vallejo-Marin 2013). Table 1 includes only those crops for which buzz pollination status could be confirmed in the literature. It should be noted that there are undoubtedly other food crops which meet the definition of being buzz-pollinated but have not yet been formally studied. For example, other *Solanum* cultivated regionally on a small scale, including *S. lapisocarpum* Dunal (Solanales: Solanaceae; the hairy-fruited eggplant) and *S. sibundoyense* Bohs (the *tomate silvestre*) are likely buzz pollinated, yet their pollination biology is undocumented. Outside of *Solanum*, the genus *Mouriri* (Myrtales: Melastomataceae) also exhibits buzz-pollination traits (poricidal anthers) and some species produce edible fruits (Buchmann and Buchmann 1981, Buchmann 1985). Specifically, the Manapuça fruit (*M. pusa* Gardner ex Gardner) is occasionally gathered from the wild and sold locally in markets in Brazil, but due to its rarity is not yet cultivated as a food crop (Lorenzi et al. 2006, Vasconcelos et al. 2010) (Table 1).

Buzz-pollinated crops produce can also have non-food uses. The Kangaroo apple or Poroporo (*Solanum aviculare* G. Forst.) is used in pharmaceuticals (Macek 1989, Weavers 2010), and the fruits of the American black nightshade (*Solanum americanum* Mill.) have been identified for their medicinal properties (Lim 2015). The scale of production for the crops in Table 1 varies, but many have been identified as promising candidates for future global fruit crops and in others, attempts have already been made to cultivate them outside of their native range (see Table 1). Understanding the pollination requirements of these minor crops now may facilitate their future exploitation.

## To What Extent Do Buzz-Pollinated Crops Rely on Pollination?

Many buzz-pollinated crops are hermaphroditic (both sexes in the same individual), and self-compatible, and even low amounts of pollen deposition on the stigma (e.g., from mechanical movement by wind), can often initiate some fertilization and fruit production (Ferguson et al. 1987, Pessarakli and Dris 2004, Kimura and Sinha 2008, Starast et al. 2012, Smreciu et al. 2013). However, this does not mean that these plants are able to fully self-pollinate autonomously and numerous studies have determined that insect pollination improves fruit set and quality in a range of buzz-pollinated crops, including in tomato (Banda and Paxton 1991), blueberry and cranberry (Isaacs and Kirk 2010, Benjamin and Winfree 2014), eggplant (Pessarakli and Dris 2004), kiwi (Miñarro and Twizell 2015), and the lulo (Almanza Fandiño 2007). Of the more limited subsample of buzz-pollinated crops examined in field studies, there is also significant evidence to suggest that pollination specifically by bees able to perform buzz pollination increases fruit yield and quality still further, e.g., in tomato (Banda and Paxton 1991), kiwi (Kim et al. 2005), blueberry and cranberry (Stubbs and Drummond 1996, Javorek et al. 2002), and eggplant (Hikawa 2004).

## Buzz Pollination in Tomato (*Solanum lycopersicum*)

The best-studied buzz-pollinated crop is the tomato (*S. lycopersicum*). Tomatoes originated in South and Central America, and their domestication and cultivation can be traced back to the early Aztecs of Mesoamerica in 700 A.D (Bergougnoux 2014). In the 16th century, colonists brought the tomato into Europe and the European colonies, and from there it spread to the rest of the world (Smith 2001, Bergougnoux 2014). Tomatoes are now found ubiquitously and there are more than 7,500 varieties, with a global annual value of USD $10.8B (Tridge 2020).

One of the most important requirements for the production of high-quality tomato fruits is pollination (Picken 1984). Tomato flowers are hermaphroditic, containing both male and female sex organs, with inflorescences that usually consist of eight to sixteen flowers at intervals of around three leaves (Picken 1984). Tomato flowers do not produce nectar and instead rely exclusively on pollen to attract and reward floral visitors. Their flowers have poricidal anthers which are fused together by small hairs to form a hollow tube or cone around the pistil and from which the stigma can be exposed to different extents (Cooper 1927, Glover et al. 2004). In some varieties, the anthers dehisce into the centre of the tube formed by the fused stamens, while in others the anthers pores point away from the flowers centre (Kaul 1991). In cases where the apex of the anther cone moves outwards, the exposed stigma is reported to be able to contact both buzzing and non-buzzing bees (Vinícius-Silva et al. 2017). Although, tomatoes are self-compatible (Free 1993), pollination is still required for full fruit set, which likely requires anther shaking from either wind, mechanical movement or insect visitation to release pollen from their poricidal anthers.

Although tomato plants in open fields are thought to be pollinated by wind-action (Hanna 1999), wind pollination alone may lead to fruits that are more likely to abort and of an inferior size and quality, compared to other pollination methods (Amala and Shivalingaswamy 2017). Interestingly, the most commonly used method of hand pollination of tomatoes is to collect pollen by simulating a bee’s vibration on the anther using a vibrating wand (Banda and Paxton 1991, Dogterom et al. 1998, Nazer et al. 2003, Cauich et al. 2004, Bell et al. 2006, Martín-Closas et al. 2006, Vergara and Fonseca-Buendía 2012, Ahmad et al. 2015). Despite its ingenuity, this method is expensive, labor intensive, and can damage the flower. It is also often less efficient than pollination by bees, both buzz pollinating and otherwise (Banda and Paxton 1991, Sabara et al. 2004, Amala and Shivalingaswamy 2017).

## Methods

### Data Collection

A literature search was carried out on Google Scholar on 30th of April 2020 using the keywords ‘Pollination, pollinator, tomatoes, tomato, *Lycopersicum* or *Lycopersicon*’, (‘allintitle: Tomato OR Tomatoes OR *Lycopersicum* OR *Lycopersicon* AND Pollination OR Pollinator’). The 381 resulting articles identified were screened to remove duplicates, articles for which we were unable to obtain the full text, articles which did not assess tomato fruit quality (fruit weight) following pollination, or those that did not have an appropriate control (no pollination). Review papers containing data not available elsewhere were included. The final list was composed of 24 articles.

Fruit weight was chosen for comparisons as weight is used in calculating crop prices, and appeared in the majority of studies, in contrast to fruit set. The experiments carried out in each study were categorized by the type of pollination treatment applied into one of five categories: 1) Buzz-pollinating bee: flowers were exposed to a buzz-pollinating bee such as *Bombus* spp.*, Xylocopa* spp. Latreille (Hymenoptera: Apidae), or *Melipona spp*. Illiger (Hymenoptera: Apidae) 2) Non-buzz-pollinating bee: flowers were exposed to a bee that is unable to produce vibrations to remove pollen from flowers, such as *A. mellifera* or *Trigona spp*. Jurine (Hymenoptera: Apidae) 3) Mechanic vibrations: flowers were exposed to artificially produced mechanical vibrations such as by a mechanical shaker, pollination wand, or electric toothbrush. 4) Auxin treatment: flowers were treated with the plant hormone auxin (Indole-acetic acid). 5) Open pollination: flowers were exposed to unmanipulated (‘natural’) pollinator assemblages in the field, including both buzz-pollinating and non-buzz-pollinating bees in unquantified proportions. Fruit weight was obtained from the text, or if not available in the text from figures using WebPlotDigitiser (Rohatgi 2017). Publications that contained multiple treatments (e.g., compared two or more different pollination methods) were entered separately in the analysis. If a single study had multiple replications of the same treatment the mean was calculated and used for analysis. The complete data set consisted of 73 experiments from 24 studies (see Supp Table S1 [online only] for full list of studies included).

In order to standardize changes in fruit weight across studies, we calculated the percentage change in fruit weight from the control (no pollination) using the equation below.

$$\begin{array}{l} \%\;change=\frac{\left(weight\;with\;SP-weight\;without\;SP\right)}{weight\;without\;SP}\times 100, \end{array}$$

where *SP (supplemental pollination) =* represents one of the five pollination treatments described above.

### Statistical Analysis

We conducted two analyses to determine the relationship between pollination treatment and fruit weight. First, we used the full data set after removing two outliers with percentage increases above 780%. The remaining 71 experiments from 24 studies were analyzed using a linear mixed-effects model with percentage change in fruit weight as the response variable, pollination treatment as a fixed effect, and study as a random effect, using the function *lmer* in the package *lme4* (Bates et al. 2014). The statistical significance of the fixed effect (treatment) was assessed using a Type III Analysis of Variance with Satterthwaite’s method using the function *anova* with the package *lmerTest* (Kuznetsova et al. 2017). The use of study as a random effect allows incorporating the statistical non-independence in cases where multiple experiments were carried out in the same study group. However, this approach does not account for the uncertainty in the estimated mean effects of individual experiments, which are affected by experimental variation and sample size. Therefore, as a second analysis we conducted a formal meta-analysis of the data. For a meta-analysis, it is necessary to obtain the sample size and variation estimate (e.g., SD or SE) associated with each mean effect reported. From the 71 experiments used in the first analysis, we excluded those that did not report the sample size and/or SD (or SE) for both the control and pollination treatments. When necessary, SD was calculated from the SE using the equation SD = SE × $\sqrt{n}$. This selection process yielded 21 individual experiments from 10 studies and did not include any Auxin treatment experiments. For the meta-analysis, we calculated standardized difference in means (Hedge’s *g*) between control and pollination treatments. Hedge’s *g* weights individual experiments based on both the SD and sample size of control and treatment means (Rosenthal 1994, Koricheva et al. 2013). We chose to use Hedge’s *g* instead of Cohen’s *d* as experiments consisted of relatively small sample sizes, *n* < 20 (Koricheva et al. 2013). The standardized mean differences were statistically analysed using linear mixed-effects models using the function *rma* in the package *metafor* (Viechtbauer 2010). Confidence intervals of coefficients which did not overlap zero were interpreted as statistically significant. All analyses were carried out in *R* ver. 4.0.2 (R Core Development Team 2020) implemented in *RStudio* (RStudio Team 2020).

## Results and Discussion

### Effect of Pollination Treatment on Tomato Weight

**Fig. 1.**
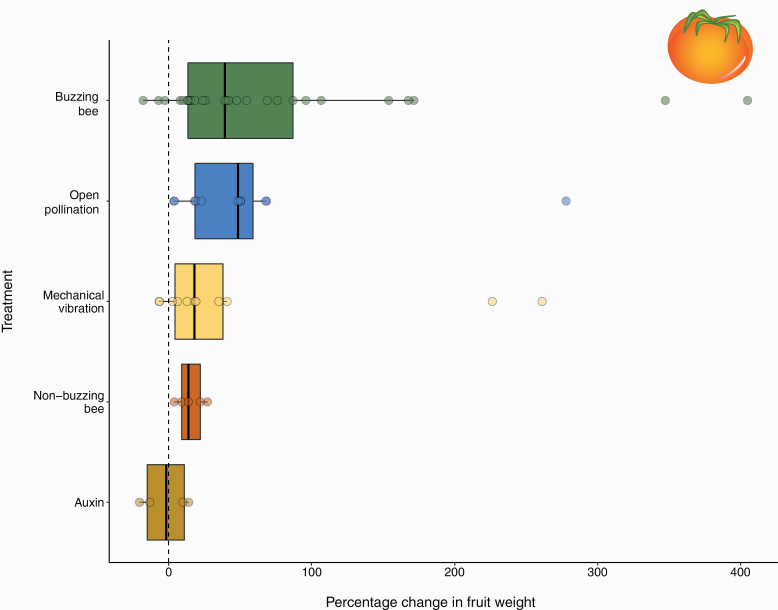
Effect of pollination treatment on percent change in tomato fruit weight compared to a no-pollination control across 71 experiments from 24 studies. Open pollination (*n* = 12), buzzing bee (*n* = 35), mechanical vibration (*n* = 13), non-buzzing bee (*n* = 6), auxin (*n* = 5). (For a full list of studies included, see Supp Table S1 [online only]).

**Fig. 2.**
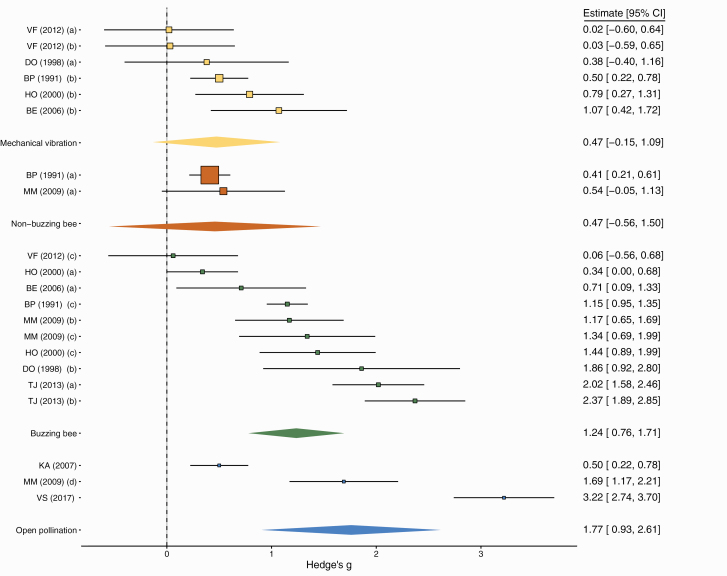
Meta-analysis of the effect of four different pollination treatments on tomato fruit weight measured as the standardized difference (Hedge’s *g*) with a no-pollination control in a subset of 21 experiments from 10 studies. Symbol size in individual studies is proportional to the weight the study has on the meta-analysis. For the unabbreviated list of studies included, see Supp Table S1 (online only).

Among the 73 experiments from 24 studies, 47.5% investigated the effect of pollination by vibration-producing bees on tomato fruit weight, 8.2% the effect of non-buzz pollinating, 18% the effect of open pollination, 21% the effect of mechanical vibrations, and 4.9% the effect of auxin application. Studies on non-buzz-pollinating bees included species in the genera *Apis, Nannotrigona* Le Peletier (Hymenoptera: Apidae), and *Trigona* (Supp Fig. S1 [online only]). Studies on buzz-pollinating bees included mostly bumblebees (*Bombus* spp.), but also species in the genera *Amegilla* Friese (Hymenoptera: Apidae), *Augochloropsis* Cockerell (Hymenoptera: Halictidae), *Exomalopsis* Spinola (Hymenoptera: Apidae), *Hoplonomia* Ashmead (Hymenoptera: Halictidae), *Melipona*, and *Xylocopa* (Supp Fig. S1 [online only]). On average, tomato flowers which received any pollination treatment produced significantly heavier fruits (effect of *Treatment F* = 5.942, df = 4, 41.86, *P* < 0.001), but this effect varied significantly among pollination treatments. We found a statistically significant increase in fruit weight in both open pollination and buzz-pollinating bee treatments on fruit weight (Fig. 1; Table 2, (A)). In contrast, auxin, mechanical vibrations, and non-buzzing bees did not significantly increase fruit weight (Table 2, (A); Fig. 1). The meta-analysis based on a subset of these experiments showed results consistent with the full analysis. The mixed-effects models on Hedge’s *g*, showed a significant overall positive effect of pollination treatment (Table 2, (B); Fig. 2). When comparing the different pollination treatments, both buzz-pollinating bees and open pollination were associated with increased fruit weight, while neither mechanic vibrations nor non-buzzing bees yielded statistically significant increases in fruit weight (Table 2, (B); Fig. 2). The effect size of open pollination varied widely, probably reflecting the heterogeneous composition of the pollinator assemblages across studies, as well as a small sample size. The effect size of mechanical vibrations and non-buzzing bees were very similar, while on average buzz-pollinating bees had a larger effect size (Fig. 2). In summary, both analyses demonstrate that the highest increase in fruit quality, measured as fruit weight, is achieved with pollination either by buzz-pollinating bees or by assemblages of bees including buzz-pollinating bees.

**Table 2.** (A) Effect of pollination treatment on tomato fruit weight measured as the percent change compared to a no-pollination control (% change). The analysis includes 71 experiments from 24 studies analysed using a mixed-effects model with study as a random effect. (B) Meta-analysis of the effect of pollination treatment in tomato fruit weight measured as the standardized difference (Hedge’s *g*) with a no-pollination control in a subset of 21 experiments from 10 studies

(A)				
Pollination treatment	Estimate	SE	*P-*value	*N* experiments
Auxin	36.40	23.16	0.121	5
Mechanical vibrations	30.13	19.41	0.129	13
Non-buzz-pollinating bees	25.20	23.24	0.283	6
Buzz-pollinating bees	64.72	17.73	**0.001**	35
Open pollination	85.37	27.81	**0.003**	12
(B)				
Pollination treatment	Estimate	SE	95% CI	*N* experiments
Mechanical vibrations	0.468	0.315	−0.148 to 1.085	6
Non-buzz-pollinating bees	0.470	0.525	−0.558 to 1.499	2
Buzz-pollinating bees	1.237	0.241	**0.764–1.710**	10
Open pollination	1.771	0.429	**0.930–2.613**	3

Confidence intervals of coefficients not overlapping zero (shown in bold) are interpreted as statistically significant.

### Factors Mediating the Effect of Bee Pollination on Tomato Yield

The studies examined here used a range of tomato varieties and cultivars, pollinated by different bee species (Supp Fig. S1 [online only]; Supp Table S1 [online only]), which may have different responses to pollination and/or attractiveness to bees. For example, Strange (2015) found a significant difference in tomato fruit weight following pollination by *Bombus huntii* between the two indeterminate varieties of tomato ‘Favorita’ and ‘Sungold’. Differences in fruit production were also noted between the indeterminate and determinate varieties of tomatoes studied in Brazil (Silva-Neto et al. 2019). The effect of pollination treatment on fruit weight could be mediated by pollinator preferences. For example, in some blueberries, different varieties can be more or less attractive to bees (Stubbs and Drummond 1996). Moreover, variation in the bee density required to achieve full pollination may also vary among tomato varieties. For example, cherry tomatoes require twice as many bumblebee colonies per hectare than beef tomatoes due to the larger number of flowers per plant (Velthuis and Van Doorn 2006). Conversely, too many colonies may lead to over visitation, and the floral damage imposed by the bee’s bite during pollination can interfere with fertilization and or lead to malformed fruits (Velthuis and Van Doorn 2006). In particular, tomato varieties with small flowers are more susceptible to damage from over visitation (Velthuis and Van Doorn 2006). Environmental factors, including temperature may also affect the capacity of bees to deliver pollination services. For instance, the buzz-pollinating stingless bee, *Melipona quadrifasciata* Le Peletier (Hymenoptera: Apidae), is thought to be an efficient tomato pollinator only when temperatures do not go above 28°C (Hikawa and Miyanaga 2009). An additional factor that could explain the varying effects of a single pollination treatment on tomato fruit weight that we observed is variation in visitation rates. Some of the studies analyzed here allowed multiple visits by bees while others only allowed a single visit. The relationship between visit number and fruit weight is unclear. While some studies indicate that a single visit is enough to achieve full seed set (Morandin et al. 2001, Nunes-Silva et al. 2013), others show that fruit weight increases with visitation rate (Hogendoorn et al. 2006). However, future studies could address the extent to which buzz-pollinating bees affect fruit yield in different tomato varieties.

We also detected considerable variation in the effect of mechanical vibrations on fruit weight across studies (Fig. 1). This variation could indicate differences among tomato varieties in the benefits of artificial vibrations. In some varieties, where the anther cone is more loose and the anther pores become almost longitudinal slits, pollen may be more readily shed even with the weak vibrations produced by wind movement, negating the benefits of applying additional vibrations (Garcia et al. 2008). In contrast, in other varieties, strong vibrations may be required to release pollen from anthers thus maximizing the benefits of applying supplemental mechanical vibrations. In addition, duration of vibrations used, number of vibrations, and vibration method varied among studies. In most of the studies, vibrating wands were used directly on the anthers (Banda and Paxton 1991, Dogterom et al. 1998, Bell et al. 2006, Hogendoorn et al. 2006, Ahmad et al. 2015). However, in other studies, vibrations were applied by using a wooden rod to hit a metal wire used as plant support causing vibrations to spread through the plants (Vergara and Fonseca-Buendía 2012). Our results suggest that the mechanism and type of vibration applied to tomato flowers may mediate the effect of mechanical vibrations on tomato quality.

### Geo-political Variation in the Use of Supplemental Buzz Pollinators for Tomatoes

The results of our systematic review and meta-analysis indicate a clear association between buzz-pollinating bees and fruit quality in tomatoes. This association supports the hypothesis that poricidal flowers, including crops such as tomatoes, achieve highest pollination when visited by bees capable of producing vibrations during floral visitation. In turn, this implies that the choice of pollinator type, and even bee species, may have important repercussions for the productivity of buzz-pollinated crops. The deployment of supplemental pollinators varies widely around the globe and is shaped by historical, economic, environmental, and even political constraints. In the remaining sections of our review, we examine the geo-political variation in the use of supplemental pollinators in tomato and highlight challenges and opportunities to improve the selection of bees used in buzz-pollinated crops grown around the globe.

### Europe: Capitalizing on *B. terrestris*

In Europe, bumblebees (specifically *B. terrestris*) are now the most popular choice of tomato pollinator. Bumblebees have been reared commercially at a relatively large scale since the late 1980s (Velthuis and Van Doorn 2006), when they subsequently replaced the more labor intensive and expensive manual pollination (Velthuis and Van Doorn 2006, Gosterit and Gurel 2018). In a seminal study, Banda and Paxton (1991) compared bumblebees (*B. terrestris*), honey bees (*A. mellifera*), and mechanical vibration (using a vibrating wand) for the pollination of greenhouse tomatoes in the United Kingdom. Their study demonstrated that manual pollination using a vibrating wand increased tomato fruit weight compared to honey bee pollination (35.9% vs 28.3% increase relative to no-pollination, respectively). However, the greatest yield increase was achieved with buzz-pollinating bumblebees, *B. terrestris* (74.5% increase in fruit weight compared to no pollination; Banda and Paxton 1991). Similarly, buzz pollination by *B. terrestris* led to significantly higher marketable fruit quality than either mechanical vibration or application of auxin spray in studies in both Spain (Martín-Closas et al. 2006) and Turkey (90% and 61% higher than mechanical vibration and auxin respectively; Daşgan et al. 2004). Therefore, in Europe, *B. terrestris* has established itself as the main supplemental pollinator of tomato crops. However, within Europe, different types of *B. terrestris* are used (Velthuis and Van Doorn 2006). For instance, although *B. terrestris* is used in most of mainland Europe, in the United Kingdom, only the local subspecies, *B. terrestris ssp. audax* Harris can be used in outdoor plots. Similarly, in the Canary Islands, only *B. terrestris ssp. canariensis* Perez is supplied by major providers of supplemental pollinators. Little is known about the efficiency of different types of bumblebees on tomato pollination, but experiments with captive bumblebees suggest that different subspecies within *B. terrestris* vary in the type of vibrations they can produce (Arroyo-Correa et al. 2019). Further work on the capacity of different sub-species and species of bumblebees to buzz pollinate might help in identifying the characteristics of pollinators with the highest potential to improve fruit yield.

### North America and Mexico: The Search for Native Pollinators

Following the success of the *B. terrestris* as a tomato pollinator in Europe, North America begun importing and deploying this bumblebee species to satisfy their own tomato pollination needs. The importation of *B. terrestris* into North America was quickly restricted due to concerns of the ecological impacts of this non-native species on local bee populations (Dogterom et al. 1998, Velthuis and Van Doorn 2006). Thus began a search for a North America native bumblebee to replace *B. terrestris* (Dogterom et al. 1998). The Common Eastern Bumblebee *B. impatiens* was originally reared for pollination in eastern North America, while the Western Bumblebee *B. occidentalis* Greene (Hymenoptera: Apidae) was reared for use in the west (Dogterom et al. 1998). Toward the end of the 1990s, *B. occidentalis*—once one of the most common bee species in North West America, underwent a rapid population decline (Colla and Ratti 2010). This decline is thought to be linked to the rapid spread of pathogens which was facilitated by international bumblebee trading (Whittington and Winston 2004, Strange 2015). Importation of colonies of *B. impatiens* to West North America was prohibited (Strange 2015) but following the rapid decline of *B. occidentalis,* emergency permits were authorized to import *B. impatiens* to make up the pollination deficit. In an effort to identify other native bumblebee pollinators, Strange (2015) compared the pollination efficiency of the commonly commercially reared *B. impatiens*, with two native western species: *B. huntii* Greene (Hymenoptera: Apidae) and *B. vosnesenskii* Radoszkowski (Hymenoptera: Apidae). Pollination by these two bumblebees increased tomato fruit weight (18.3% and 13.7%, respectively) compared to no pollination, and both were deemed suitable alternatives to *B. impatiens* in North West America. Nevertheless, mass rearing protocols have not yet been developed for these species. Incidents such as the rapid decline of *B. occidentalis* highlight the danger of relying on a single species of commercial pollinator and emphasize the importance of maintaining pollinator diversity for ensuring robust and resilient pollination services (Sabara et al. 2004).

*Bombus impatiens* does not naturally occur in Mexico, but colonies of this species have been imported there for tomato pollination since 1994 (Velthuis and Van Doorn 2006). As in other regions, concerns of introducing non-native species have encouraged attempts to transition to native bees. One early candidate was the stingless, non-buzz-pollinating bee *Nannotrigona perilampoides* Cresson (Hymenoptera: Apidae) (Palma et al. 2008). Although *N. perilampoides* performed better than mechanical vibration in experimental trials, they were outperformed by *B. impatiens* in terms of tomato yield and quality (Palma et al. 2008). Among Mexico’s bumblebees, the primary candidate of interest for tomato pollination is *B. ephippiatus* Say (Hymenoptera: Apidae), which has demonstrated a pollination efficiency on tomatoes comparable with *B. impatiens* (Torres-Ruiz and Jones 2012, Vergara and Fonseca-Buendía 2012). An ongoing barrier against the widespread use of *B. ephippiatus* is the increased difficulty of mass-rearing colonies. Hence, bumblebee pollination still relies on non-native *B. impatiens*, despite the fact this introduced species has been identified as a threat to Mexican bumblebees (Vergara and Fonseca-Buendía 2012). The continued use of commercially reared, non-native pollinators remains a matter of serious concern for the maintenance of local bee diversity.

Mexico has a large number of buzz-pollinating native bees and their contribution to tomato pollination has been assessed previously (Macias-Macias et al. 2009). The two most abundant native bee taxa found in this study were solitary, buzz-pollinating bees *Examalopsis* spp. (Apidae) and *Augochloropsis* spp.. Visitation by *Exomalopsis* spp. alone removed 20% of pollen grains from tomato flowers, compared to just 5% by honey bees. Pollination by *Exomalopsis spp*. also led to a significantly higher tomato fruit weight (47.9%) and number of seeds (150.1%) compared to no pollination. Similarly, *Augochloropsis spp*. removed 19% of pollen grains, and significantly increased fruit weight (54.55%) and number of seeds (158.67%) compared to no pollination (Macias-Macias et al., 2009). Despite their efficiency and the high abundance of *Exomalopsis* spp. and *Augochloropsis* spp., fruit quality was significantly higher in tomato plants in the open pollination plot than from pollination by either of these species alone. The superiority of fruit produced from the open plot, where flowers were potentially visited by a broader assemblage of bees emphasizes the importance of bee diversity rather than just abundance in tomato pollination (Macias-Macias et al. 2009).

### South America: Bumblebees and Other Native Pollinators

*Bombus terrestris* was first imported from Europe to Chile in 1998 for tomato pollination, and importation continues to this day, despite evidence of invasion and anti-importation legislation in surrounding countries (Velthuis and Van Doorn 2006, Aizen et al. 2019). The introduction and rapid establishment of *B. terrestris* in Chile and Argentina is associated with the decline of native bumblebees, including the largest bumblebee in the world, *B. dahlbomii* Guerin-Meneville (Morales et al. 2013)*. Bombus atratus* Franklin has been identified as a native bumblebee with the potential to replace *B. terrestris* for tomato pollination in South America. Preliminary studies in both Colombia and Uruguay found that tomato pollination by *B. atratus* significantly increased yield and quality of tomato fruit compared to both no pollination and auxin application (Cure and Rodríguez 2007, Salvarrey et al. 2020). These are hopeful results, and efforts toward the mass rearing of *B. atratus* have begun, and already reared colonies can be purchased on a small scale (Padilla et al. 2017).

Research on tomato pollination in Brazil has clearly recognized the opportunity to capitalize on wild assemblages of native bees (Franceschinelli et al. 2013, Vinícius-Silva et al. 2017, Roubik 2018). Looking beyond bumblebees, Silva-Neto et al. (2019) investigated the potential of the native buzz-pollinating stingless bee *M. quadrifasciata* Le Peletier (Hymenoptera: Apidae) for greenhouse tomato pollination in Brazil. *Melipona quadrifasciata* pollination generates fruits of higher quality than no pollination (Silva-Neto et al. 2019). Pollination by this stingless bee also results in significantly larger and higher quality tomato fruit compared to *A. mellifera* pollination (dos Santos et al. 2009). Interestingly, in this case, pollination by *A. mellifera* led to fruit that was the same weight and size as those which had received no pollination (dos Santos et al. 2009), which could be seen as indicative of the limited benefits of using non-buzz pollinators. However, other studies on bee diversity in open fields of tomatoes in Brazil have found that even when *Melipona* bees are present in the area they are not generally observed in tomato fields, and it has also been suggested that the short overlap period between *Melipona* foraging activity and tomato stigma receptivity lowers their efficiency as pollinators (Del Sarto et al. 2005, Macias-Macias et al. 2009). A potential solution to this problem might be to capitalize on South America’s rich bee fauna, which includes many buzz pollinators. Field tomato crops which are accessible to native pollinating bees have a significantly greater yield and quality of fruit than those which exclude a varied range of native pollinators (Franceschinelli et al. 2013, Deprá et al. 2014, Santos et al. 2014, Vinícius-Silva et al. 2017, Roubik 2018). Again, these studies highlight the importance of maintaining and capitalizing upon a diverse portfolio of pollinators even in the case of a relatively specialized buzz-pollination system.

### Australasia: The Threat of *B. terrestris* and the Use of Native Bees

A number of British bumblebees were imported to New Zealand for red clover pollination in 1885 and 1906, and several of these species have since become established (Hopkins 1914). Consequently, *B. terrestris* is now reared commercially in New Zealand for tomato pollination (Velthuis and Van Doorn 2006). However, tomato pollination in Australia is almost entirely reliant on manual mechanical vibration (Bell et al. 2006). A large reason for this is that, apart from feral invasive bumblebees in Tasmania, Australia has no native bumblebee species (Hingston 2006). In order to remain competitive with imported tomato prices, farmers have put pressure on the Australian government to allow the importation of commercial bumblebee colonies for supplemental pollination (Bell et al. 2006). However, the potential ecological risks of importing bumblebees to Australia remain very high (Hergstrom et al. 2002, Griffiths 2004).

Several studies have attempted to find an alternative tomato pollinator within Australia’s own bee fauna (Hogendoorn et al. 2000, 2006, 2007; Bell et al. 2006). Potential candidates have included the buzz pollinating: green carpenter bee *Xylocopa lestis* Smith, and the blue-banded bees *Amegilla holmesi* Rayment and *Amegilla chlorocynea* Cockerell. These preliminary studies demonstrated that pollination by *X. lestis*, *A. holmesi*, and *A. chlorocynea* lead to increases in tomato fruit weight (13.56%, 12.91%, and 72.22%, respectively) compared to no pollination (Hogendoorn et al. 2000, 2006; Bell et al. 2006). Pollination by blue-banded bees was also directly compared to pollination by mechanical vibration. While *A. holmesi* produced fruit with a slightly lower weight, *A. chlorocynea* produced tomatoes of higher weight than those pollinated mechanically (Hogendoorn et al. 2000, 2007; Bell et al. 2006). Interestingly, Hogendoorn (2010) also determined that tomatoes pollinated by the buzz-pollinating blue banded bee *Amegilla murrayensis* Rayment (Hymenoptera: Apidae) were significantly tastier than the manually pollinated tomatoes (Hogendoorn et al. 2010)! Despite the potential that blue-banded bees and carpenter bees demonstrate as tomato pollinators in Australia, their utilization on large scales is currently impractical for a variety of reasons, including the bees’ incompatibility with Australian greenhouse design, reluctance to take pollen from a dish, and an inability to rear them in large enough numbers (Hogendoorn et al. 2000, 2007; Bell et al. 2006). Further research to overcome the practical challenges involved in using native Australian buzz-pollinating bees seems timely and particularly urgent.

### Asia: Searching the Balance for Commercially Reared Pollinators

Despite concerns from ecologists, in 1991, the European bumblebee, *B. terrestris* was imported to Japan for tomato pollination (Ono 1998). Unsurprisingly, *B. terrestris* quickly became invasive, displacing and competing with native Japanese bumblebees (Inoue et al. 2008). Following evidence of this negative impact, and heated debate between ecologists and farmers, *B. terrestris* importation to Japan was prohibited in the late 2000s (Goka 2010). The notion of using native Japanese bumblebees for tomato pollination had not been overlooked, and Asada and Ono (1996) emphasized that 9 of the 15 native bumblebee species in Japan had successfully been reared in the laboratory and could therefore be cultivated as native pollinators. Asada and Ono (1996) examined tomato fruit quality following pollination by the Japanese bumblebees *B. ardens* Smith, *B. diversus* Smith, *B. hypocrita* Perez, *B. ignitus*, and non-native imported *B. terrestris*. Japanese bumblebees were shown to be as efficient as the imported bee at pollinating tomato crops, significantly increasing tomato fruit yield compared to no pollination. In actuality, the rearing of these bees proved difficult (Asada and Ono 1996). Although *B. ignitus* is now reared commercially in Japan for greenhouse tomato pollination, colonies are much smaller and have a narrower foraging range meaning considerably more colonies are needed per hectare (Velthuis and Van Doorn 2006).

Mass reared native bumblebees, however, may not be the safety net ecologists had hoped for. Commercially reared native bees produced on a mass scale with low genetic diversity could still increase the spread of bee pathogens, as well as influence genetic diversity of their wild counterpart if/when they interact with native bees during foraging or mating (Hikawa and Miyanaga 2009). To circumvent these problems, Hikawa and Miyanaga (2009) suggest a radical new strategy: importing *Melipona* stingless bees from the neotropics for tomato pollination in Japan. *Melipona* bees are found throughout the warm areas of the Neotropics and colonies have been cultivated by humans for over 3,000 yr in Mesoamerica (Quezada-Euán 2018). Hikawa and Miyanaga (2009) suggest that using *Melipona* bees in tomato greenhouses in Japan would not pose a risk of species invasion, as *Melipona* thermoregulatory ability means they cannot overwinter in temperate zones. They subsequently tested the pollination efficiency of *M. quadrifasciatus* in comparison to *B. terrestris*, finding that overall tomato fruit yield and quality was comparable between both species, apart from at low floral pollen levels where *B. terrestris* pollinated flowers produced a significantly greater yield and quality of tomatoes (Hikawa and Miyanaga 2009). To our knowledge, the suggestion of using neoptropical buzz pollinators for tomato pollination in temperate zones has not yet been implemented.

Like Japan, in Taiwan, there have been some restrictions on *B. terrestris* importation for tomato pollination, due to concerns of ecological invasion (Sung and Chiang 2014). Farmers in Taiwan have historically used plant growth regulators like auxins to encourage fruiting and yield; however, the process of application is costly and time consuming (Chen and Hanson 2001). Research has therefore turned to non-buzz-pollinating honey bees which are already commonly reared throughout the region, as well as to Taiwan’s native bumblebees. Sung and Chiang (2014) found that pollination by native bumblebee *B. eximus* Smith resulted in significantly better fruit than was achieved by either honey bee pollination or auxin application. Despite its suitability and efficiency, commercial rearing of *B. eximu*s is difficult as it requires large initial stocks of native bees, which is a challenge due to their steep native terrain, as well as the potentially damaging effect that taking bees from wild populations could have (Sung and Chiang 2014). Sung and Chiang conclude that if advanced precautionary measures were implemented, that included extending quarantine and established control measures, *B. terrestris* should be imported from abroad to maintain tomato fruit yield and quality (Chen and Hanson 2001, Sung and Chiang 2014).

In Indonesia, tomato pollination is often left to either wind pollination, or honey bees are brought into supplement pollination (Putra and Kinasih 2014). Putra et al. (2014) argues that due to their low climate adaptability and invasive nature, neither imported honey bees nor bumblebees are suitable pollinators under tropical conditions (Putra and Kinasih 2014). In order to address this, Putra et al. investigated the pollination efficiency of wild Indonesian honey bee *Apis cerana* Fabricius and local stingless bee *Trigona iridipennis* Smith, as pollinators of tomatoes in open-field conditions. Both of these bee species are already cultivated and managed domestically for their honey and wax products, so colonies are readily available for transport and establishment (Putra and Kinasih 2014). However, neither of these bees are capable of buzz pollination, and total fruit production per plant and quality was found to be only marginally higher, or the same, under pollination by either bee species, compared to no pollination, with Asian honey bee pollination slightly outperforming that of the stingless bee. Both bees were also noted to preferentially visit other plants making visitation rates low, although *T. iridipennis* was reported to have a somewhat higher floral constancy than *A. cerana* (Putra and Kinasih 2014). Researchers in India have also investigated the efficiency of their wild bees as tomato pollinators, with Amala et al. in 2017 investigating the potential of buzz-pollinating bees *Amegilla zonata* L. (a blue banded bee) and *Hoplonomia westwoodi* Gribodo (Hymenoptera: Halictidae; a sweat bee) for pollination of field-grown tomatoes. Unlike the non-buzz pollinators in Indonesia pollination by both buzz-pollinating bees resulted in significantly heavier tomato fruits than with no pollination (154% and 87%, respectively; Amala and Shivalingaswamy 2017).

In Pakistan, in order to reduce the labor and time costs associated with manual tomato pollination, Ahmad et al. 2015 identified *B. terrestris* as an efficient pollinator of two varieties of tomato, leading to significantly increased fruit quality and yield in both varieties, compared to no pollination and pollination by manual vibration (Ahmad et al. 2015). Research is now being undertaken to rear native Pakistani bumblebee *B. haemorrhoidalis* Richards for tomato pollination (Sharma et al. 2018). Likewise, in Israel, efforts have focused on rearing native subspecies *B.* terrestris ssp. dalmatinus Dalla Torre for tomato pollination in the region (Velthuis and Van Doorn 2006). Whereas, in Jordan, Nazer et al. found that *B. terrestris* pollination led to significantly greater tomato fruit quality and yield compared to both plant growth regulators and mechanical vibration, and recommended their use in tomato pollination (Nazer et al. 2003).

### Africa: Potential Benefits of Bee Pollinators

Fewer studies have investigated tomato pollination in Africa, despite the fact that they are considered a commercially important crop in the region (IPBES 2016). The distribution of *B. terrestris* extends as far as coastal Northern Africa where it is used for pollination purposes. However, south of the Sahara no bumblebees occur naturally and the importation of *B. terrestris* is not permitted (Velthuis and Van Doorn 2006). Across the continent managed pollinators are little used (Toni et al. 2020), and research has focused instead on encouraging farmers to capitalize on native pollinators. A study in Ghana in 1990 found that fruit set was significantly higher for those plots open to insect visitors compared to plots caged with honey bees alone, although interestingly fruit volume and weight were higher from plants caged with honey bees (Amoako and Yeboah-Gyan 1990). Another study, in Kenya reported that tomato plots open to native pollinators achieved a significantly higher fruit quality than those where pollinators were excluded (Kasina 2007). The main visitors to tomato flowers in this study were the buzz pollinators *Xylcopa calens* Le Peletier and *Halictus spp*. which were observed releasing pollen via buzz pollination. Visitation by *A. mellifera* was also reported, however here these non-buzz pollinators were observed to tear and damage anthers to obtain pollen, and considered robber species (Kasina 2007). Both *Xylocopa spp*. and *A. mellifera* have also been observed on tomato plots in other countries of the region (Choudourou et al. 2012, Toni et al. 2020) These studies are concordant with a previous report that acknowledged that tomatoes specifically are likely to benefit from a native assemblage of bees in African countries (2016). As highlighted further in Toni et al (2020), clearly, more work on tomato pollination in Africa is urgently needed to determine the extent to which tomato production can be improved via supplemental pollination (Toni et al. 2020).

### The Role of Diversity in Buzz Pollination

The results from our systematic review and metanalysis of pollination in tomato indicate a high average increase in fruit weight following pollination by both individual buzz pollinator species and native assemblages of bees, which in every study investigated included a variety of buzz pollinators. Thus, our study joins others emphasizing the importance of pollinator diversity rather than abundance in improving the yield and quality of crops (Klein et al. 2003, Hoehn et al. 2008, Albrecht et al. 2012, Winfree et al. 2018). In areas with a rich pollinator diversity, the best and most practical pollinators may be the native assemblages readily available, and efforts should be made to protect and promote them (Macias-Macias et al. 2009, Franceschinelli et al. 2013). Evidence from studies in tomatoes (Kasina 2007, Macias-Macias et al. 2009, Franceschinelli et al. 2013, Vinícius-Silva et al. 2017, Gaglianone et al. 2018), blueberries (Stephen et al. 2008, Tuell et al. 2009, Isaacs and Kirk 2010, Scott et al. 2016), and eggplants (Gemmill-Herren and Ochieng 2008, Montemor and Malerbo Souza 2009, Desuó 2014, Mainali et al. 2018) suggest that buzz-pollinated crops often benefit from a diversity of buzz-pollinating bees. One reason for this could be that a diversity in size of different buzz-pollinating bee species complements the diversity in flower size found in tomato plants For example, it has been suggested that in some buzz-pollinated flowers, the size matching between the flower’s reproductive organs and bee size can yield increased seed set (Solis-Montero and Vallejo-Marin 2017). Moreover, a wide range of visitors which are active at different times of the day may help ensure that floral visitation coincides with pollen availability and stigma receptivity which can vary throughout the day (Kaul 1991). Finally, an array of buzz-pollinating bee species might exhibit different vibrational characteristics (e.g., vibrations of a different frequency and/or amplitude). If the relationship between vibrational properties and pollen release varies among buzz-pollinated flowers, thus a diverse portfolio of buzz pollinators provides greater opportunity for optimal matching between flowers and buzz-pollinating bees whose ‘buzz’ is best suited to remove pollen from a particular buzz-pollinated flower (King and Buchmann 2003, De Luca et al. 2013). An assemblage of buzz-pollinating bees may, therefore, be able to maximize pollination by improving both pollen removal and deposition.

Encouraging a diversity of bee species may not only be beneficial in its own right but also prevents the super exploitation of a particular species and the issues associated with this (Dafni et al. 2010), as well as providing buffer species to guarantee pollinator presence should one species fare poorly in any given year (Santos et al. 2014). Utilizing native assemblages of bees in buzz-pollinated crops would include considering species specific requirements of buzz pollinators that make up the local fauna. For example, it is important to consider the nesting requirements of different bee species and the relative importance of proximity to natural habitats (Greenleaf and Kremen 2006). Consideration should also be taken to the floral resource requirement of different bee species throughout the year and how this can be capitalized upon. Further efforts to encourage native bees should include preservation of native fragments near cultivated areas, pollinator informed agrochemical application and conservation of soil, and prevention of erosion for ground nesting bees (Greenleaf and Kremen 2006, Gaglianone et al. 2018).

The results from studies on native bee assemblages, as well as numerous studies emphasizing the importance of diversity over abundance (Klein et al. 2003, Hoehn et al. 2008, Albrecht et al. 2012, Winfree et al. 2018), open an intriguing avenue of thought as to the potential benefits of supplementing pollination with colonies from more than one species of bee. Currently, it seems studies have only investigated combinations of bumblebees and honey bees and their effect on tomato fruit yield or quality. Although one study found that the combination of honey bees and bumblebees led to a significantly reduced fruit weight compared to bumblebees alone (Nazer et al. 2003), another study found no significant difference between bumblebee pollination alone, and bumblebee and honey bee pollination (Higo et al. 2004). Further work is clearly needed. Perhaps by taking inspiration from the specific assemblages of native bees found together in the wild, researchers could identify combinations of pollinators that might work together. Furthermore, pollinator assemblages could be designed by considering aspects of their foraging, for example phenological activity patterns, to ensure pollinator coverage over most of the flowering time, with minimal pollinator competition.

## Conclusions

Pollination of buzz-pollinated crops often relies on managed bees, both native and non-native. Our results show that in terms of crop productivity, buzz-pollinating bees have an edge in increasing yield (fruit weight) of buzz-pollinated crops compared to pollination by mechanical means or through non-buzz-pollinating bees. The history of supplemental pollination for tomato crops alone has been fraught with complications and concerns associated with the overuse of non-native and or/managed bee species. We suggest that when managed pollination is required, priority should be placed into developing and employing native bee species. However, even native bees can be detrimental to wild bee populations, and recent studies illustrate how dominant managed species such as honey bees can displace wild bee populations within their native range (Herrera 2020). Therefore, care must be taken to ensure that managed buzz pollinators (e.g., commercial *Bombus* spp. colonies) do not become a problematic resource in their native range (Mallinger et al. 2017). The next frontier in sustainable pollination of buzz-pollinated crops lies in the use of wild populations of bees. Such an approach will require more fundamental changes in practices, including those that preserve the habitat complexity and floral diversity required to sustain a diverse bee community (Venturini et al. 2017, Requier and Leonhardt 2020). Buzz-pollinated plants and their bee pollinators represent a tangible example of the importance of considering bee functional diversity in the pollination of both wild and agricultural species.

## Supplementary Material

toab009_suppl_Supplementary_Table_S1

toab009_suppl_Supplementary_Figure_S1
