# Supplementary material for: Buzz-Pollinated Crops: A Global Review and Meta-analysis of the Effects of Supplemental Bee Pollination in Tomato
Source: J Econ Entomol. 2021 Feb 22;114(2):505–19. doi: 10.1093/jee/toab009 (PMC8042731; doi:10.1093/jee/toab009)
Supplement: toab009_suppl_Supplementary_Table_S1 [file toab009_suppl_supplementary_table_s1.pdf]

Buzz-Pollinated Crops: A Global Review and Meta-analysis of the Effects of Supplemental Bee Pollination in Tomato

Hazel Cooley & Vallejo-Marin
Journal of Economic Entomology

Table S1. Full list of 73 experiments and 24 studies included arranged by percentage change in fruit weight relative to a no-pollination control (% Change in fruit weight). ID = Experiment number. For full references see the Reference section in the article's main text. Studies included in the meta-analysis are indicated with *.

ID	Study	Cultivation type	Location	Tomato variety	Treatment	% Change in fruit weight
**1**	Martin-Closas Puigdomenech & Pelacho 2007	Greenhouse	Spain	Bond	Auxin	-20.281
**2**	Strange 2015	Greenhouse	USA	Sungold	Buzzing bee	-18.074
**3**	Martin-Closas Puigdomenech & Pelacho 2007	Greenhouse	Spain	Bond	Buzzing bee	-17.791
**4**	Martin-Closas Puigdomenech & Pelacho 2007	Greenhouse	Spain	Bond	Auxin	-13.201
**5**	Salvarrey et al. 2020	Greenhouse	Uruguay	Elipida	Auxin	-12.172
**6**	Martin-Closas Puigdomenech & Pelacho 2007	Greenhouse	Spain	Bond	Buzzing bee	-6.983
**7**	Martin-Closas Puigdomenech & Pelacho 2007	Greenhouse	Spain	Bond	Mechanic	-6.667
**8**	Martin-Closas Puigdomenech & Pelacho 2007	Greenhouse	Spain	Bond	Mechanic	-6.320
**9**	Vergara & Fieldonseca-Buendia 2012*	Greenhouse	Mexico	NA	Mechanic	2.841
**10**	Gaglianone et al. n.d.	NA	Brazil	Salada	Open pollination	3.661
**11**	Putra & Kinasih 2014	Field	Indonesia	NA	Non-buzzing bee	3.939
**12**	Gaglianone et al. n.d.	NA	Brazil	Cherry	Open pollination	4.167
**13**	Vergara & Fieldonseca-Buendia 2012*	Greenhouse	Mexico	NA	Mechanic	5.405
**14**	Dogterom Matteoni & PlowriGreenhouset 1998*	Greenhouse	Canada	BeeFieldsteak	Mechanic	6.137
**15**	Strange 2015	Greenhouse	USA	Fieldavorita	Buzzing bee	6.419

**16**	Silva-Neto et al. 2019	Greenhouse	Brazil	Santa Cruz	Buzzing bee	8.119
**17**	Cauich et al. 2004	Greenhouse	Mexico	Maya	Non-buzzing bee	9.076
**18**	Vergara & Fieldonseca-Buendia 2012*	Greenhouse	Mexico	NA	Buzzing bee	10.187
**19**	Bell Spooner-hart & HaiGreenhouse 2006*	Greenhouse	Australia	NA	Buzzing bee	12.911
**20**	Cauich et al. 2004	Greenhouse	Mexico	Maya	Mechanic	12.966
**21**	Strange 2015	Greenhouse	USA	Sungold & Fieldavorita Cherry Tomato	Buzzing bee	13.303
**22**	Hogendoorn Steen & Schwarz 20008*	Field	Australia	Grosselissi	Buzzing bee	13.559
**23**	Strange 2015	Greenhouse	USA	Sungold & Fieldavorita Cherry Tomato	Buzzing bee	13.660
**24**	Putra & Kinasih 2014	Field	Indonesia	NA	Non-buzzing bee	13.826
**25**	Nazer Kasrawi & Al-Attal 2003	Greenhouse	Jordan	NA	Auxin	14.023
**26**	Silva-Neto et al. 2019	Greenhouse	Brazil	AP533 hybrid	Buzzing bee	15.109
**27**	Bell Spooner-hart & HaiGreenhouse 2006*	Greenhouse	Australia	NA	Mechanic	18.043
**28**	Kasina 2007*	Field	Kenya	NA	Open pollination	18.085
**29**	Strange 2015	Greenhouse	USA	Sungold & Fieldavorita Cherry Tomato	Buzzing bee	18.297
**30**	Gaglianone et al. n.d.	NA	Brazil	Aguamiel	Open pollination	19.004
**31**	Nazer Kasrawi & Al-Attal 2003	Greenhouse	Jordan	NA	Mechanic	19.122
**32**	Strange 2015	Greenhouse	USA	Sungold	Buzzing bee	19.200
**33**	Banda & Paxton 1991*	Greenhouse	England	BeeFieldsteak	Non-buzzing bee	19.539
**34**	Macias-Macias Chuc Ancona-Xiu Cauich & Quezada-Eua 2009*	Field	Mexico	Saladette	Non-buzzing bee	22.193
**35**	Gaglianone et al. n.d.	NA	Brazil	Chipano	Open pollination	23.077

**36**	Salvarrey et al. 2020	Greenhouse	Uruguay	Lapataia	Buzzing bee	23.844
**37**	Dogterom Matteoni & PlowriGreenhouset 1998*	Greenhouse	Canada	BeeFieldsteak	Buzzing bee	25.684
**38**	Strange 2015	Greenhouse	USA	Fieldavorita	Buzzing bee	26.091
**39**	Salvarrey et al. 2020	Greenhouse	Uruguay	Elipida	Auxin	29.874
**40**	Banda & Paxton 1991*	Greenhouse	England	BeeFieldsteak	Mechanic	30.500
**41**	Hogendoorn 2006*	Greenhouse	Australia	NA	Mechanic	36.163
**42**	Banda & Paxton 1991*	Greenhouse	England	BeeFieldsteak	Non-buzzing bee	37.010
**43**	Salvarrey et al. 2020	Greenhouse	Uruguay	Elipida	Buzzing bee	37.736
**44**	Cure and Rodriguez 2007	Greenhouse	Colombia	Durinta	Buzzing bee	40.900
**45**	Banda & Paxton 1991*	Greenhouse	England	BeeFieldsteak	Mechanic	41.277
**46**	Salvarrey et al. 2020	Greenhouse	Uruguay	Elipida	Buzzing bee	41.331
**47**	Nazer Kasrawi & Al-Attal 2003	Greenhouse	Jordan	NA	Buzzing bee	42.068
**48**	Macias-Macias Chuc Ancona-Xiu Cauich & Quezada-Eua 2009*	Field	Mexico	Saladette	Buzzing bee	47.594
**49**	Gaglianone et al. n.d.	NA	Brazil	Sweet gold	Open pollination	48.551
**50**	Fieldranceschinelli et al. 2013	Field	Brazil	Italian	Open pollination	50.212
**51**	Gaglianone et al. n.d.	NA	Brazil	Italian	Open pollination	50.212
**52**	Macias-Macias Chuc Ancona-Xiu Cauich & Quezada-Eua 2009*	Field	Mexico	Saladette	Buzzing bee	54.545
**53**	Banda & Paxton 1991*	Greenhouse	England	BeeFieldsteak	Buzzing bee	62.612
**54**	Macias-Macias Chuc Ancona-Xiu Cauich & Quezada-Eua 2009*	Field	Mexico	Saladette	Open pollination	67.914
**55**	Gaglianone et al. n.d.	NA	Brazil	Sophia	Open pollination	68.541
**56**	Hogendoorn 2006*	Greenhouse	Australia	NA	Buzzing bee	69.019
**57**	Banda & Paxton 1991*	Greenhouse	England	BeeFieldsteak	Buzzing bee	86.458
**58**	Amala & Shivalingaswamy 2017	Field	India	S. lycopersicum	Buzzing bee	87.017
**59**	Hikawa & Miyanaga 2009	Greenhouse	Japan	NA	Buzzing bee	87.629

**60**	Hikawa & Miyanaga 2009	Greenhouse	Japan	NA	Buzzing bee	91.753
**61**	Torres-Ruiz & Jones 2013*	Greenhouse	Mexico	Tricia	Buzzing bee	96.039
**62**	Torres-Ruiz & Jones 2013*	Greenhouse	Mexico	Tricia	Buzzing bee	106.780
**63**	Amala & Shivalingaswamy 2017	Field	India	S. lycopersicum	Buzzing bee	154.042
**64**	Hikawa & Miyanaga 2009	Greenhouse	Japan	NA	Buzzing bee	158.696
**65**	Hikawa & Miyanaga 2009	Greenhouse	Japan	NA	Buzzing bee	167.391
**66**	Ahmad et al. 2015	Greenhouse	Pakistan	Cherry	Mechanic	226.316
**67**	Vinicius-Silva et al. 2017*	Field	Brazil	NA	Open pollination	227.777
**68**	Ahmad et al. 2015	Greenhouse	Pakistan	Grandella	Mechanic	261.165
**69**	Vinicius-Silva et al. 2017*	Field	Brazil	NA	Open pollination	344.223
**70**	Ahmad et al. 2015	Greenhouse	Pakistan	Cherry	Buzzing bee	347.368
**71**	Ahmad et al. 2015	Greenhouse	Pakistan	Grandella	Buzzing bee	404.854
**72**	Hikawa & Miyanaga 2009	Greenhouse	Japan	NA	Buzzing bee	783.333
**73**	Hikawa & Miyanaga 2009	Greenhouse	Japan	NA	Buzzing bee	900.000
