# Supplementary material for: Buzz-Pollinated Crops: A Global Review and Meta-analysis of the Effects of Supplemental Bee Pollination in Tomato
Source: J Econ Entomol. 2021 Feb 22;114(2):505–19. doi: 10.1093/jee/toab009 (PMC8042731; doi:10.1093/jee/toab009)

Buzz-Pollinated Crops: A Global Review and Meta-analysis of the Effects of Supplemental Bee Pollination in Tomato

Hazel Cooley & Vallejo-Marin
Journal of Economic Entomology

Figure S1. Effect of supplemental pollination on tomato fruit weight relative to a no-pollination control. MV = Mechanical vibration. Non-buzzing bees: *Apis, Nanotrigona, Trigona*. Buzzing bees: *Amegilla, Augochloropsis, Bombus, Exomalopsis, Hoplonomia, Melipona.*

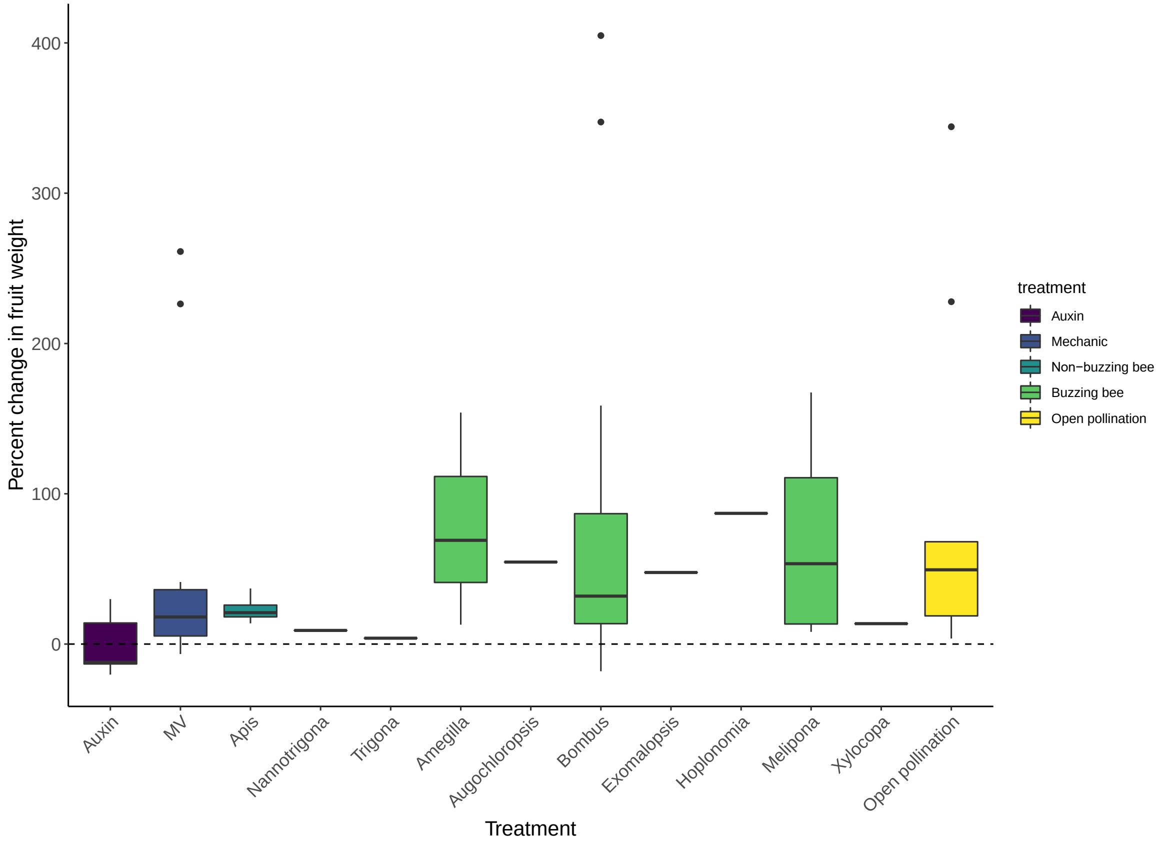

Supplement: toab009_suppl_Supplementary_Figure_S1 [file toab009_suppl_supplementary_figure_s1.pdf]
